# More than a key—the pathological roles of SARS-CoV-2 spike protein in COVID-19 related cardiac injury

**DOI:** 10.1016/j.smhs.2023.03.004

**Published:** 2023-03-30

**Authors:** Zhiqiang Lin

**Affiliations:** Masonic Medical Research Institute, 2150 Bleecker Street, Utica, NY, 13501, USA

**Keywords:** COVID-19, Spike protein, Cardiovascular disease, Immune responses

## Abstract

Cardiac injury is common in hospitalized coronavirus disease 2019 (COVID-19) patients and cardiac abnormalities have been observed in a significant number of recovered COVID-19 patients, portending long-term health issues for millions of infected individuals. To better understand how Severe Acute Respiratory Syndrome Coronavirus 2 (SARS-CoV-2, CoV-2 for short) damages the heart, it is critical to fully comprehend the biology of CoV-2 encoded proteins, each of which may play multiple pathological roles. For example, CoV-2 spike glycoprotein (CoV-2-S) not only engages angiotensin converting enzyme II (ACE2) to mediate virus infection but also directly activates immune responses. In this work, the goal is to review the known pathological roles of CoV-2-S in the cardiovascular system, thereby shedding lights on the pathogenesis of COVID-19 related cardiac injury.

## List of abbreviations:

COVID-19: coronavirus disease 2019
CoV: coronavirus
SARS-CoV-2 (CoV-2): Severe Acute Respiratory Syndrome Coronavirus 2
CoV-2-S: Severe Acute Respiratory Syndrome Coronavirus 2 spike protein
ARDS: acute respiratory distress syndrome
PRR: pattern recognition receptor
ACE2: angiotensin converting enzyme II
SAg: superantigen
APC: alternative complement activation pathway
DAMPs: danger associated molecular patterns
MI: myocardial infarction
TMPRSS2: transmembrane serine proteinase 2

## Introduction

In 2003, severe acute respiratory syndrome coronavirus (SARS-CoV) caused SARS epidemic in Asia^1^; in 2012, the first case of Middle East Respiratory Syndrome (MERS) was reported,^2^ marking the outbreak of MERS epidemic in the Arabian Peninsula. Since 2019, the coronavirus disease 2019 (COVID-19) pandemic has put the whole world in danger. Behind all these two epidemics and one pandemic is one group of viruses, the coronavirus (CoVs) family.

CoVs are enveloped RNA viruses. Until now, 7 CoVs have been known to infect humans, including HCoV-229E, HCoV-NL63, HCoV-OC43, HCoV-HKU1, SARS-CoV, MERS-CoV, and SARS-CoV-2.^3^ The first four CoVs only cause “common cold” or minor upper/lower respiratory diseases.^4^^,^^5^ In contrast, SARS-CoV, MERS-CoV, and SARS-CoV-2 are deadly viruses that cause acute respiratory distress syndrome (ARDS) and death.^3^ SARS-CoV-2 is the pathogen of COVID-19, and its genome shares an 82% identity with that of SARS-CoV.^3^ In addition to high genome sequence similarity, SARS-CoV-2 and SARS-CoV share similar pathogenesis patterns, and so most of the knowledge about SARS-CoV can be referenced to understand the pathogenesis of SARS-CoV-2.

Since the outbreak of COVID-19, scientists and front-line health workers have been trying their best to understand this syndrome. As a result of their endeavors, the death rate of COVID-19 has been greatly reduced and a series of excellent reviews about the pathology of COVID-19 have been published,^6^, ^7^, ^8^, ^9^, ^10^ which are invaluable for those readers who want to understand more about COVID-19.

Over the course of the COVID-19 pandemic, cardiovascular injury is common in hospitalized COVID-19 patients and associated with increased mortality,^11^ and persistent cardiac injury is also common among COVID-19 survivors.^12^ To treat acute and post-acute COVID-19 manifestations, significant efforts need to be put forth to dissect the pathogenesis of COVID-19-related cardiovascular diseases. Here I reviewed the molecular mechanism of how SARS-CoV-2 damages the cardiovascular system, with a focus on elucidating the multiple pathological roles of SARS-CoV-2 spike protein in COVID-19-associated cardiovascular diseases.

## The pathophysiology of myocardial dysfunction in COVID-19

**Fig. 1:**
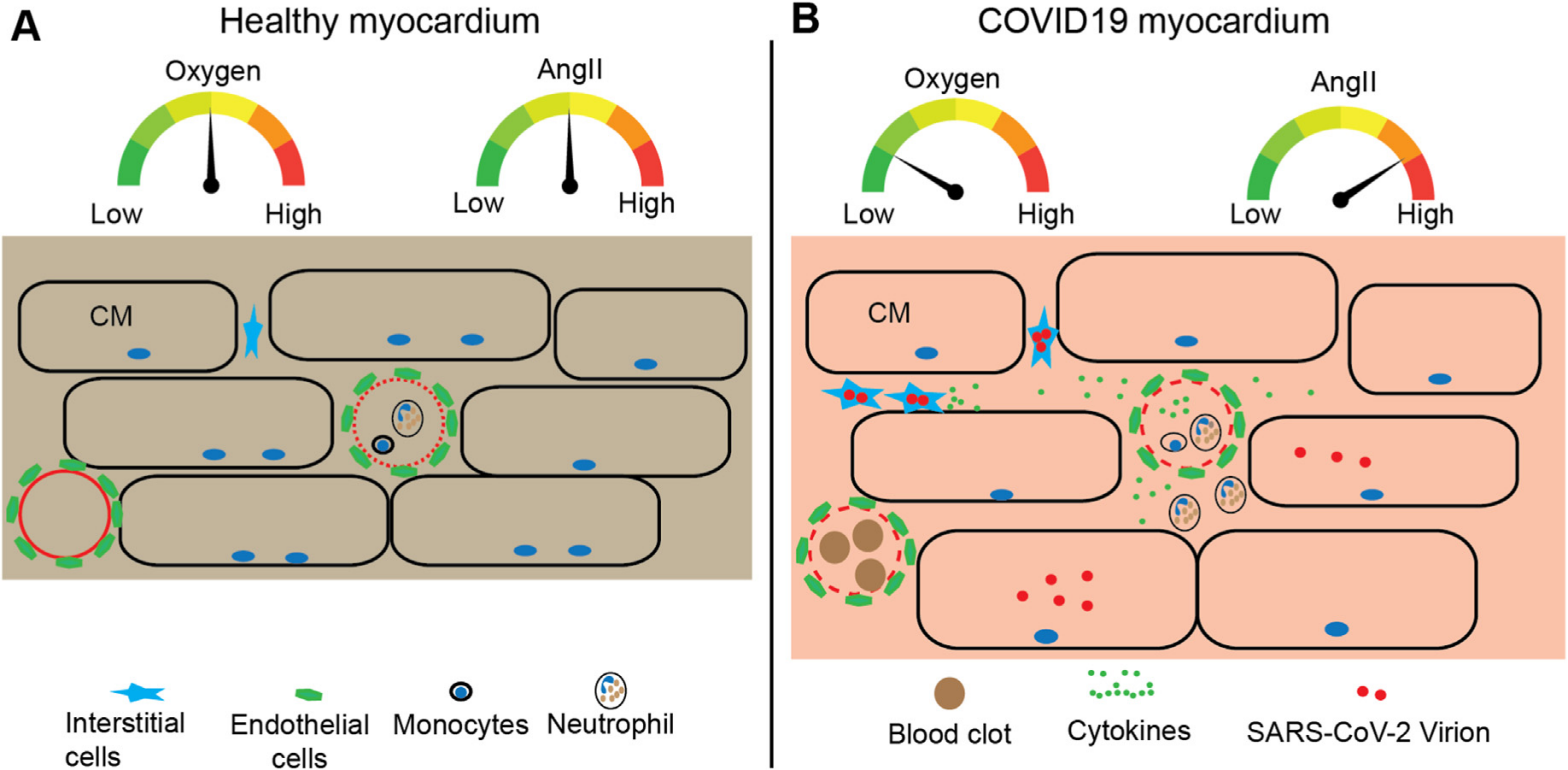
**Comparison of normal and COVID-19 stressed myocardium. A**. Normal myocardium. **B**. COVID-19 stressed myocardium with edema. Patients with severe COVID-19 symptoms are at high risk of developing heart injury. Their heart muscle layers are facing multifaceted stresses, including but not limited to hypoxia stress caused by acute respiratory distress syndrome (ARDS) and coronary artery micro-thrombosis, imbalanced neurohormones (e.g. Ang II), cytokine storm stress, and SARS-CoV-2 infection-caused CM loss.

Contagious infection of SARS-CoV-2 (CoV-2) may result in severe illness and bring overwhelming stress to the circulatory system. Therefore, it is anticipated that COVID-19 patients with underlying cardiovascular conditions are prone to have severe adverse outcomes, which has been confirmed by two independent retrospective cohort studies^11^^,^^13^ and one recently published prospective longitudinal study.^14^ These studies also singled out cardiac injury as one independent risk factor associated with a higher mortality rate in hospitalized COVID-19 patients. Additionally, independent cardiac magnetic resonance imaging studies clearly showed cardiomegaly in athletic COVID-19 patients.^7^^,^^15^^,^^16^ In this review, by combing through the clinical observations and pathological findings, I highlighted four reasons that may cause myocardial injury and cardiac dysfunction in COVID-19 patients (Fig. 1).

### CoV-2 infection causes hypoxemia stress

For each beat, the heart needs a large amount of oxygen to generate power for pumping blood throughout the whole body. Therefore, low blood oxygen saturation (known as hypoxemia) will cause hypoxia stress to the heart and reduce heart function. Severe COVID-19 patients usually have acute lung injury, which may progress to ARDS and severe hypoxemia^17^ which causes cardiac damage. Serologic cardiac troponin T (cTnT) level is a standard diagnose marker for cardiac injury^18^ and COVID-19 patients with elevated cTnT levels also had severe respiratory dysfunction,^11^ therefore, bolstering the causal relationship between hypoxemia and cardiac injury. Besides ARDS-induced hypoxemia, COVID-19-caused coronary microthrombi also contribute to myocardial injury,^19^ and electrocardiograms (ECGs) of COVID-19 patients with cardiac injury displayed abnormalities that are compatible with myocardial ischemia, such as T-wave depression and inversion, ST-segment depression and pathological Q-waves.^13^^,^^19^

One COVID-19 case study clearly demonstrated a causal relationship between hypoxemia and cardiac dysfunction. In this study, a 69-year-old male patient had worsening dyspnea before being hospitalized, and he quickly developed end-stage heart failure (% Ejection Fraction ​= ​25%) and severe hypoxemia after being registered in the hospital. After receiving Extracorporeal membrane oxygenation (ECMO) treatment that provides respiratory support, the patient's left ventricle function progressively recovered to a normal range on day 5,^20^ thus suggesting that hypoxemia is one of the primary reasons causing cardiac injury in hospitalized COVID-19 patients.

### CoV-2 infection induces neurohumoral stress

The renin-angiotensin system (RAS) plays central roles in maintaining blood pressure homeostasis.^21^ In this system, renin is an aspartic protease that cleaves liver-synthesized angiotensiongen to produce angiotensin (an inactive decapeptide hormone), which is further converted to angiotensin II (AngII, an active octapeptide vasoconstrictive hormone) by angiotensin converting enzyme (ACE). As a homologue of ACE, ACE2 converts AngII to Ang 1–7. AngII has pleiotropic biological effects, such as increasing vessel contraction, activating renal sympathetic nerve cells to release catecholamine hormones, promoting the generation of reactive oxygen species (ROS), and enhancing inflammatory responses.^22^ On the contrary Ang1-7 mediates anti-inflammatory and vasodilatory effects.^23^

Acute stress-induced cardiomyopathy, also known as Takotsubo cardiomyopathy, is a reversible heart failure syndrome that resembles acute myocardial infarction (MI). In these patients, the coronary arteries are not obstructed, and characteristic transient ballooning of the apical and mid-ventricular myocardium is presented.^24^^,^^25^ This symptom is triggered by emotional or physical stress, and clinical manifestations include myocardial macrophage inflammatory infiltration,^25^ increase of systemic pro-inflammatory cytokines and circulating catecholamine levels.^26^

CoV-2 uses ACE2 as its receptor to infect human cells,^27^ which harms the human body by killing two birds with one stone: on one hand, the wide expression of ACE2 makes the virus easy to spread; on the other hand, engaging and inactivating ACE2 potentially predisposes the patients to neurohumoral stresses by increasing AngII level.^28^ In a COVID-19 case study, a 43-year-old female patient had oppressive chest pain, dyspnea, and oxygen desaturation. Lab test results showed elevated cTnT, NT-proBNP, and C-reactive protein, indicating the occurrence of cardiac injury. Echocardiography data showed mild left ventricular systolic dysfunction and anterolateral ventricle wall hypokinesis. No ventricle dilation and pericardial effusion were detected. The patient was diagnosed with acute virus-negative lymphocytic myocarditis, and her cardiac systolic function fully recovered after receiving anti-virus medicine treatment.^29^ This report suggests that dramatic mental or physical stresses caused by CoV-2 may elicit Takotsubo cardiomyopathy in COVID-19 patients.

### SARS-CoV-2 infection induces septic stress

Sepsis is a systemic inflammatory response to severe and acute pathogen infection.^30^ The pathophysiology of septic stress involves complicated interactions between pathogens and the host innate immune system that recognizes pathogens through pathogen-associated molecular patterns (PAMPs), such as lipopolysaccharide (LPS) in bacteria, and DNA or RNA in viruses. The battle between pathogens and host cells generates pro-inflammatory cytokines and danger-associated molecular patterns (DAMPs).^31^ PAMPs and DAMPs can be recognized by host cell pattern recognition receptors (PRRs), which are specialized signaling proteins capable of initiating innate immune responses to curtail pathogens' spread.^32^ In an infected tissue, like a domino reaction, the DAMPs released by damaged cells bind to PRRs localized on healthy cells and elicit the production of more pro-inflammatory cytokines and even lead to the generation of reactive oxygen and nitrogen species to further damage the tissue.^33^

In severe COVID-19 patients, CoV-2 infection causes tremendous damage to the lung^34^^,^^35^ and also leads to endotoxemia by disrupting the integrity of the gastro-intestinal barrier.^36^ Elevated levels of pro-inflammatory cytokines and increased neutrophil to lymphocytes ratio are two common features in these patients,^37^ indicating the high chance of septic stress. Septic stress-induced cardiovascular dysfunction is associated with a high mortality rate in sepsis patients.^38^ It is well-known that septic stress/shock causes myocardial dysfunction by affecting the hemodynamics, blood coagulation, vascular permeability, and cardiomyocyte (CM) contraction.^39^ The early phase of septic stress is characterized by high cardiac output and low peripheral vascular resistance, and the late phase comprises hypotension, poor peripheral perfusion, and low cardiac output.^39^ In the sepsis-stressed myocardium, pro-inflammatory cytokines cause endothelial dysfunction and cardiac vascular leakage, followed by a series of harmful events, such as myocardial edema^40^ and neutrophils infiltration.^41^ Meanwhile, pro-inflammation cytokines and DAMPs directly bind to their receptors on the CMs to induce apoptosis or dysfunction.^42^ All these detrimental septic insults together hinder proper myocardium function.

### CoV-2 infection leads to viral myocarditis

The aforementioned pathological insults, such as hypoxia stress, neurohumoral stress, and sepsis stress, all can lead to myocarditis, a cardiac inflammatory disease defined by inflammatory infiltrates and non-ischemic myocardial injury. Based on the nature of cardiac inflammation, myocarditis is categorized as viral infection-induced myocarditis and nonviral myocarditis.^43^

Recently, two independent clinical studies demonstrated that myocardial injury was frequent not only in severely ill COVID-19 patients but also in critically ill non-COVID-19 patients, suggesting that cardiac involvement is not a specific feature of COVID-19.^44^^,^^45^ On top of these clinical observations, post-mortem pathological studies suggest that microthrombi^19^ is one of the primary causes of myocardial injury, lending additional support to the notion that nonviral myocarditis is the predominant form of cardiac injury in severely ill COVID-19 patients.

Although rare, CoV-2 does infect the heart and causes acute viral myocarditis, at a rate of 0.2%–0.4% among hospitalized COVID-19 patients.^46^ In these CoV-2 viral myocarditis patients, CoV-2 antigens and RNA were detected in the CMs^47^, ^48^, ^49^ and cardiac interstitial cells,^20^ confirming that viral myocarditis is one of the causes of COVID-19-associated heart diseases.

In summary, this section described four possible stresses leading to cardiac dysfunction. Although these stresses were discussed separately, it is important to keep in mind that they may happen simultaneously in severely ill COVID-19 patients. Since CMs are the basic cardiac functional units and are adversely affected by COVID-19 stresses, in the next section, I will focus on discussing the possible molecular mechanisms that underly CM damage in COVID-19.

## The molecular mechanisms leading to CM injury in COVID-19

**Fig. 2:**
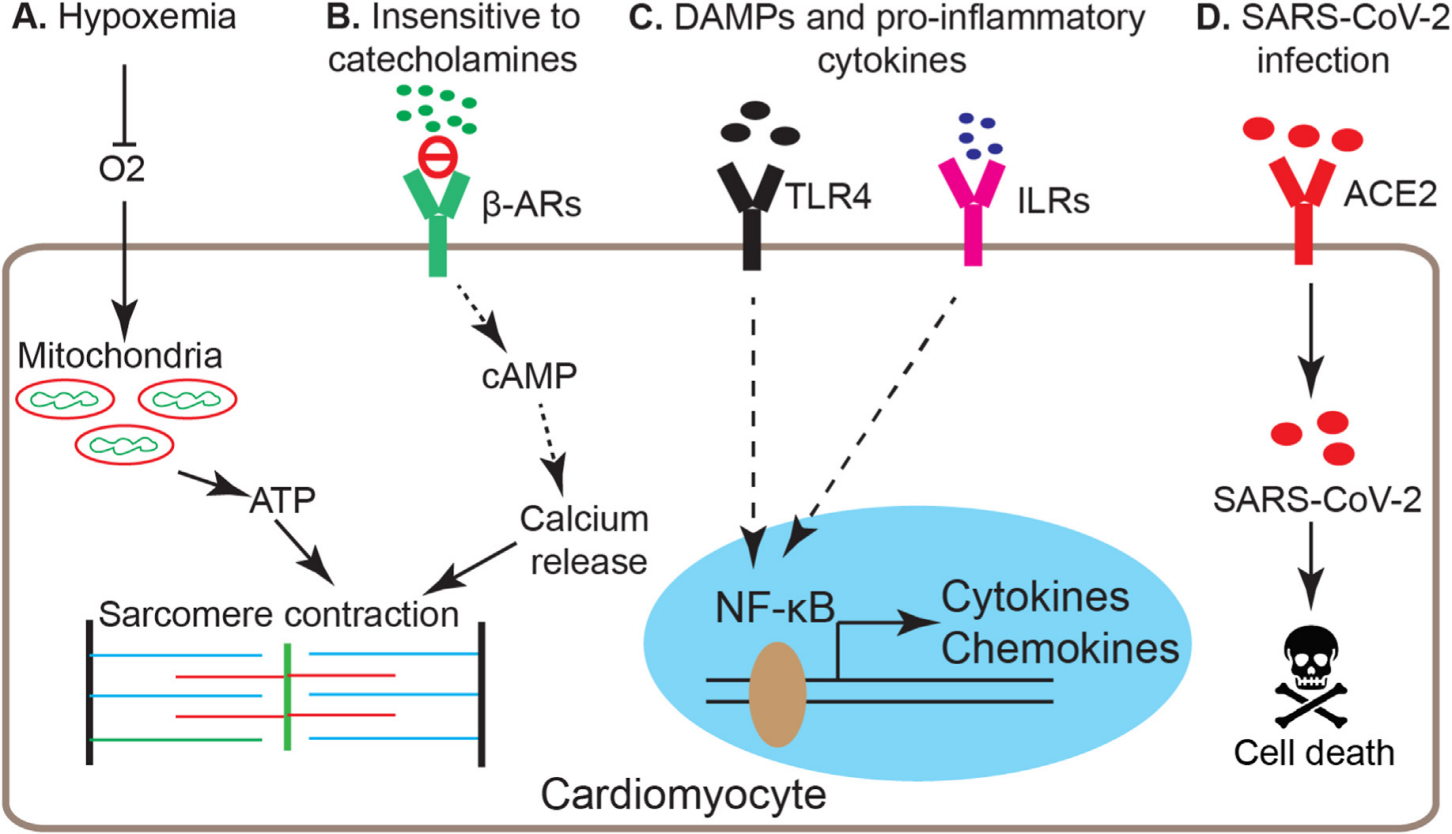
**Pathological insults that damage the** cardiomyocytes **(CMs) in COVID-19 patients.** The CMs are the functional units of the heart, and their loss or dysfunction is the root of cardiac injury. Under homeostasis condition, the CMs are well-supplied with oxygen and their behavior is oscillated by catecholamine hormones. In the myocardium stressed by COVID-19, the CMs are short of oxygen (A) and become insensitive to catecholamines (B). Additionally, circulating pro-inflammatory cytokines or danger associated molecular patterns (DAMPs) damage the CMs by activating their innate immune responses (C). In the worst scenario, SARS-CoV-2 directly infects and destroys CMs (D).

The heart contains multiple cell types including CMs, endothelial cells, fibroblasts, neuron cells, and immune cells, and all of these cell types are under stress in the COVID-19 heart. The dysfunction of different cardiac cells may all contribute to COVID-19-related heart injury, which has been nicely outlined in a recent review by Nishiga et al.^50^ In this section, I will focus on summarizing the possible cellular and molecular mechanisms that lead to CM injury (Fig. 2).

### Hypoxia stress damages the CMs

As mentioned above, hypoxemia is a common feature of severely ill COVID-19 patients.^17^ Long-time hypoxemia is detrimental and causes cardiac dysfunction.^51^ On the cellular level, mitochondria respiration generates ATP for the CMs, whose synchronized and constant beating powers the heart. Under the shortage of oxygen supply, the CMs switch to anaerobic glycolysis and generate ATP by turning glucose into lactate. Accumulation of lactate in the CMs increases cellular osmotic gradients, which in turn causes CM edema or apoptosis.^52^^,^^53^ Shortage of oxygen also causes the mitochondria to ROS,^54^ a spectrum of highly active chemicals that damage cell membranes, DNA, organelles, and induce apoptosis.^55^ In summary, the CMs can temporarily handle the hypoxia stress, but prolonged hypoxia will lead to CM dysfunction and death (Fig. 2A).

### β-adrenergic receptor desensitization impairs CM function

The heart beating rate is established by the Sinoatrial Node (SAN), a cluster of specialized pacemaker CMs localized in the right atrium.^56^ By influencing the SAN cells, the sympathetic and parasympathetic nerves increase and decrease heart rate, respectively. On the molecular level, the beating rhythm of pacemaker cells is controlled by the hyperpolarization-activated, cyclic nucleotide–gated channels (HCNs) that are responsible for the hyperpolarization-activated current (I_f_).^57^ Catecholamines are neurotransmitters synthesized by sympathetic neurons and chromaffin cells in the adrenal medulla. Catecholamine molecules, such as epinephrine, norepinephrine, and dopamine, signal the CMs to increase heart rate and contractility. On the CM surface, epinephrine activates β-adrenergic receptors (β-AR) to promote the synthesis of cyclic nucleotides (e.g., cAMP). cAMP in turn initiates signal cascades to increase calcium release^58^ and I_f_ current,^59^^,^^60^ which boosts the heart muscle contractility and pacemaker cell beating rate, respectively (Fig. 2B).

The CMs have evolved a desensitization mechanism to avoid continuous activation of β-AR signaling. Overactivation of β-AR desensitizes the β-AR by inducing β-AR phosphorylation and internalization.^58^ Because CoV-2 infection can potentially increase the adrenergic tone by engaging ACE2 and by inducing neurohumoral stress, it is likely that the desensitization of β-AR partially accounts for cardiac dysfunction in COVID-19 patients.

### DAMPs and cytokines/chemokines activate CM innate immune responses

CoV-2 infection can cause massive lung tissue damage,^34^ which provokes the release of DAMPs, such as HMGB1, DNA, cell debris, mitochondria, etc, and these DAMPs can potentially be brought to the heart through the circulatory system. Under homeostasis conditions, the CMs express low levels of Toll like receptors (TLRs), a family of pattern recognition receptors (PRRs) that recognize DAMPs. Under disease conditions, such as ischemia and hypertension, the CMs increase TLRs expression^61^ and therefore become vulnerable to DAMPs molecules, which bind TLRs and elicit signal cascades that promote the production of pro-inflammatory cytokines, and even cause cell death (Fig. 2C). For example, activation of TLR4 is detrimental to the heart, because TLR4 and its downstream kinases promote cytokine genes expression,^62^ and may also cause pyroptosis,^63^ a caspase-1 dependent cell death process characterized by cell membrane leakage and release of DAMPs.^64^ On the contrary, loss of TLR4 is beneficial and protects the heart against lipopolysaccharide (a bacteria-derived toxin) stress.^65^

CoV-2 infection also causes pro-inflammatory cytokine storm, and the pro-inflammatory cytokines have multiple adverse effects that directly or indirectly cause CM apoptosis and dysfunction. In the heart, the CMs are supported by a fine-tuned microenvironment: oxygen and nutrients are delivered through capillary vessels; sympathetic and parasympathetic nerves provide connections between the CMs and the other parts of the body; residential innate immunes cells guard the CMs against pathogens invasion. The increase of pro-inflammatory cytokines disrupts this microenvironment by interfering with the function of endothelial cells, nerve cells, and innate immune cells. For instance, IL-1β promotes coagulation^66^ and vessel permeability^67^; TNFα signals sympathetic nerves to increase the inotropic tone^68^; IL-17 helps recruit inflammatory neutrophils into the myocardium.^69^

Pro-inflammatory cytokines also directly change CMs behavior through the cytokine receptors expressed on CM surface.^70^ Once bound and activated by the pro-inflammatory cytokines, these receptors initiate maladaptive gene expression programs that lead to CM apoptosis, hypertrophy, and dysfunction. In COVID-19 patients, multiple numbers of pro-inflammatory cytokines are known to elevate to form a cytokine storm that stresses the internal organs, including the heart. These up-regulated pro-inflammatory cytokines include IL-6, IL-1β, IL-2, IL-8, IL-17, G-CSF, GM-CSF, IP10, MCP1 and MIP1α,^8^ many of which are detrimental to the heart. For a better understanding of which cytokines might get involved in COVID-19 cardiac injury, the cellular and molecular effects of the listed pro-inflammatory cytokines were summarized in Table 1.

**Table 1:** The cellular and molecular effects of cytokines elevated in severe COVID-19 patients^a^.

Name	Function in immune system	Effects on the heart and CMs	Clinical trials
IL-1b	Promote the activity of the innate immune cells.	Promotes ER stress-induced apoptosis.^72^	Summarized in a recent excellent article.^76^
	Required for T_H_17 ​cells differentiation and maintenance.^71^	Reduces L-type Ca^2+^ current.^73^	
		Increases diastolic SR Ca^2+^ leak.^74^	
		Promotes cytoskeleton rearrangement and mitochondrial alterations.^75^	
IL-6	Has broad effects on immune cells and nonimmune cells.	Loss of IL-6 attenuates cardiac inflammation.^78^^,^^79^	Summarized in a recent excellent article.^82^
	Has context-dependent pro- and anti-inflammatory properties.^77^	Protects CMs against acute oxidative stress.^80^ Decreases CM contractility.^81^	
		Promotes cytoskeleton rearrangement and mitochondrial alterations.^75^	
IL-8	Recruits and activates neutrophils in inflammatory regions.^83^	Increases adhesion of neutrophils to CMs *in vitro*.^85^ Promotes cytoskeleton rearrangement and mitochondrial alterations.^75^	Phase I, HuMax-IL8, the monoclone antibody of IL-8, safe for patients with solid tumor.^86^
	May regulate angiogenesis, tumorigenesis and fibrosis.^84^		
IL-17	Regulates the expansion and recruitment of neutrophils.	Increases CM apoptosis.^87^	Secukinumab, the monoclone antibody of IL-17, effective for psoriasis.^88^
	Promotes the expression of cytokines and anti-microbial genes in nonimmune cells, such as fibroblasts and endothelial cells.^69^		
GCSF	Important for proliferation and differentiation of haematopoietic cells; Regulates the proliferation and function of Tregs.^89^	Attenuates isoproterenol induced cardiac hypertrophy.^90^	Recombinant GCSF therapy decreases left ventricle systolic function.^92^
		Increases CM hypertrophy *in vitro*. Increases angiogenesis and decrease fibrosis in a murine MI model.^91^	
GMCSF (CSF2)	Similar with GCSF.	Helps recruit leukocytes into injured hearts. Loss of GMCSF protects MI injured hearts from rupture.^93^	No significant adverse effects, but failed to improve survival of sepsis patients.^95^
		Priming cardiac inflammation in a murine Kawasaki disease model.^94^	
IP10 (CXCL10)	Induces chemotaxis, apoptosis.	Overexpression of IP10 results in less cardiac damage and better cardiac function in coxsackievirus induced myocarditis model.^97^	Phase II, Anti-IP-10 antibody is safe but not effective for ulcerative colitis.^98^^,^^99^
	Inhibits cell growth and angiogenesis.^96^		
MCP1 (CCL2)	Regulates migration and infiltration of monocytes/macrophages.^100^	Loss of MCP1 attenuates MI-induced cardiac remodeling.^101^^,^^102^	Propagermanium is clinically used CCL2/CCR signaling inhibitor.^103^
MIPa (CCL3)	Regulates CD8^+^ T Cell function and migration.^104^	Loss of MIPa mitigates cardiomyopathy in a murine Chagasic disease model.^106^	Not available.
	Required for recruiting neutrophils to virus infected tissue.^105^		
IL-2	Required for Treg cell differentiation, survival, and function.^107^	IL-2 indirectly causes heart depression.^108^	Summarized in an excellent article.^107^

a , abbreviations used in this table. IL, interleukin; T_H_17, T helper type 17 ​cells, characterized by production of IL-17; Treg, regulatory T lymphocytes; ER, endoplasmic reticulum; SR, sarcoplasmic reticulum; GCSF, granulocyte colony-stimulating factor; GMCSF, granulocyte-macrophage colony-stimulating factor; IP10, Interferon gamma-induced protein 10, also known as C-X-C motif chemokine ligand 10; MCP1, monocyte chemoattractant protein-1, also known as Chemokine (CC-motif) ligand 2 (CCL2); MIPa, macrophage inflammatory factor 1a, also known as CCL3; MI, myocardial infarction.

### SARS-CoV-2 infects and damages CMs

The CoV-2 life cycle includes seven crucial steps: attachment, entering host cells, uncoating, gene expression, replication, assembly, and release. The first two steps are mediated by CoV-2 spike glycoprotein (CoV-2-S) and its host receptor ACE2, therefore ACE2 expression pattern determines CoV-2 tissue tropism.^109^ Protruding out of the virus membrane, CoV-2-S latches to host cells through ACE2.^21^ After attaching to host cells via CoV-2-S/ACE2 interaction, CoV-2 invades the host cells through either cell membrane fusion pathway or clathrin-mediated endocytosis pathway.^110^

In the past three years, accumulating data have confirmed that CoV-2 infects both CMs and cardiac interstitial cells,^20^^,^^111^, ^112^, ^113^, ^114^ and CoV-2 infection of CMs have been recapitulated in both animal models and cell culture systems. For example, intranasal delivery of CoV-2 to hamsters caused cardiac inflammation,^115^^,^^116^ and CoV-2 virions were detected in CMs including the pacemaker cells.^116^^,^^117^ *In vitro*, CoV-2 efficiently infects hiPSC-derived CMs (hiPSC-CMs)^49^^,^^116^^,^^118^, ^119^, ^120^; however, it has very low infection efficiency on cardiac pericytes,^121^ hiPSC-derived fibroblast,^49^^,^^118^ and hiPSC-derived endothelial cells.^122^ After successful infection, CoV-2 harms the CMs in a plethora of different ways, such as inducing the formation of syncytia,^123^ breaking down the contractile machinery,^120^ impairing electrical functions,^119^ and eventually resulting in cell death^117^ (Fig. 2D). Furthermore, whole genome sequencing data from several independent groups all show that CoV-2 infection of hiPSC-CMs induces robust innate immune responses.^49^^,^^116^^,^^118^, ^119^, ^120^^,^^124^ For understanding more details about the mechanisms of how CoV-2 damages CMs, please refer to a recently published review by Qian group.^125^

## The pathological roles of CoV-2-S in COVID-19

To better understand how CoV-2 damages the heart, it is critical to fully comprehend the biology of CoV-2 encoded proteins, each of which may have multiple pathological roles. CoV-2 genome encodes 13 functional protein-coding genes, including four structural genes: spike surface glycoprotein (S), envelope protein (E), membrane glycoprotein (M), nucleocapsid phosphoprotein (N); two polyprotein genes ORF1a, ORF1ab; and 7 accessary protein genes: ORFs 3a, 3c, 6, 7a, 7b, 8, 9b.^126^ The protein product of ORF1a is polyprotein pp1a, which can be proteolytically cleaved into 11 mature non-structural proteins named nsp1-11. Similarly, the protein product of ORF1ab is pp1ab, and its 15 cleaved products are named nsp1–10 and nsp12–16.^126^ Over the past three years, researchers have made great advances toward understanding the pathological roles of CoV-2 accessary proteins, and the results have been summarized in a recent review.^127^

During the infection process, CoV-2-S serves as a key to unlocking host cells and therefore determines CoV-2 tropism and infection efficiency.^27^ CoV-2-S has two subunits, S1 and S2, connected by a unique protein convertase site (PPC) that does not exist in other human CoVs.^128^ Before binding to ACE2, CoV-2-S needs to be cleaved at the PPC site to expose its receptor binding domain that resides in the S1 subunit. Once encountering host cells, CoV-2 infects them through two different paths: the cell membrane fusion route and the endocytosis route.

In the cell membrane fusion route, transmembrane serine proteinase 2 (TMPRSS2) from the host cells cleaves S2 subunit and causes it to undergo dramatic structural change, which allows the cleaved S2 (S2’) to insert into the host cell membrane and facilitates the forming of virus-host cell fusion pores through which the CoV-2 RNA and RNA-associated nucleocapsid are released into the host cells.^129^

In the endocytosis route, clathrin, a triskelion-shaped scaffold protein that acts to form an endosome, is essential for virus infection.^130^ Once engulfed by clathrin-mediated phagocytosis, the viral particles release their RNA into host cell cytosol by forming fusion pores on endosomal membranes, a process that requires Cathepin L-mediated CoV-2-S cleavage.^131^^,^^132^ Of note, CoV-2 may primarily take advantage of the endocytosis route to infect CMs, because blocking Cathepin L and not TMPRSS2 activity abolished CoV-2 infection of hPSC-derived CMs.^49^

**Fig. 3:**
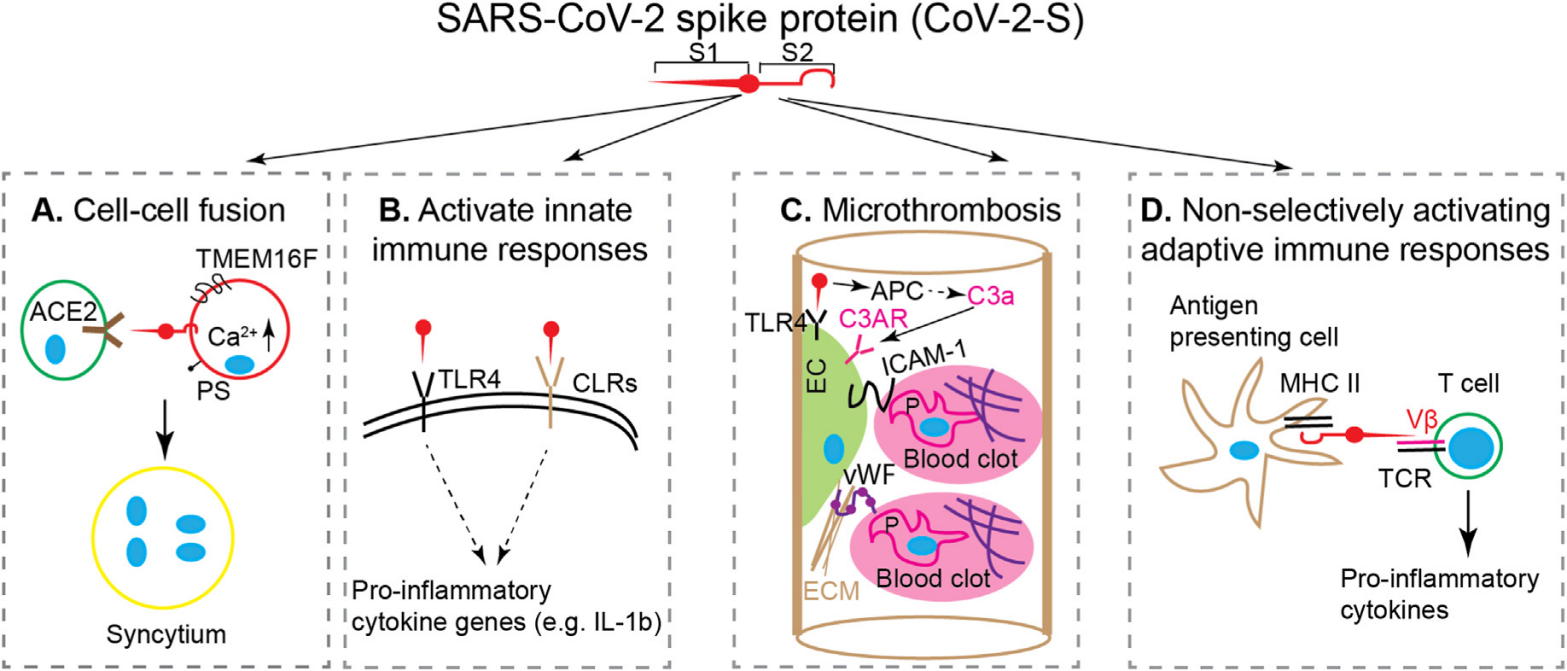
**SARS-CoV-2 spike protein (CoV-2-S) has multiple pathological roles.** Besides serving as the crucial factor mediating virus entry, CoV-2-S also harms the host cells and organs in many ways. **A.** CoV-2-S glues neighboring cells together to form a giant cell containing multiple nuclei (Syncytium). PS: phosphatidylserine. **B.** CoV-2-S binds to cell surface-localized innate immune receptors to inflame tissue cells and peripheral immune cells. TLR4, Toll-like receptor 4; CLRs, C-type lectin receptors. **C.** CoV-2-S damages the vascular endothelial cells and activates the complement system, leading to the formation of microthrombi in small vessels. EC, endothelial cell; ECM, extracellular matrix; C3AR, Complement Component 3a Receptor; ICAM-1, intercellular adhesion molecule; vWF, von Willebrand factor; P, platelet; APC, alternative complement activation pathway. **D.** CoV-2-S cross links antigen presenting cells and T cells, activating a large T cell population (2%–20%) by skipping the antigen selection process. MHC II, Major histocompatibility complex class II; TCR, T cell receptor; Vβ, TCR β chain variable domain.

Due to its importance in the CoV-2 life cycle, CoV-2-S has gained attentions from many researchers and a large body of publications has shown that in addition to facilitating virus entry, CoV-2-S also has other pathological roles that govern CoV-2 virulence. In the following sections, I will focus on updating the non-infection pathological roles of CoV-2-S, with an aim of extending our knowledge about whether CoV-2 employs its spike protein to damage the cardiovascular system (Fig. 3).

### CoV-2-S causes cell-cell fusion

A hallmark of CoV-2 infection is the formation of syncytium (plural, syncytia), a giant cell that contains multiple nuclei.^133^ Specifically, the presence of pneumocytes syncytia is one of the typical pathological characteristics of deceased COVID-19 patients.^134^ This is because CoV-2 infection can force the neighboring host cells to fuse together (Fig. 3A).

The formation of syncytia is not a unique feature of COVID-19. Under normal physiological conditions, cell-cell fusion events do happen to accomplish a specialized biological function, such as myoblast fusion during skeleton muscle growth, epithelia fusion at the time of placenta formation, and macrophages fusion during tissue remodeling.^135^ Phosphatidylserine (PS) represents only a small fraction of the plasma membrane phospholipid components and its distribution is asymmetric, being exclusively retained in the cytofacial leaflet of the plasma membrane under homeostasis status.^136^ One of the crucial steps of cell-cell fusion is PS exposure which makes the cells fusion-competent. PS exposure is enzyme facilitated process, during which PS is translocated from the cytofacial leaflet to the exofacial surface,^136^ and this PS translocation event is probably performed by calcium (Ca^2+^)-activated phospholipid scramblases, such as Transmembrane Protein 16F (TMEM16F).^137^

In CoV-2 infected cells, CoV-2-S awakes the normally silenced cell-fusion machinery and forces these cells into a fusion-competent status, as evidenced by a recent study by Braga et al. who reported that expressing CoV-2-S in Vero Cells (an epithelial cell line) induced sudden Ca^2+^ transient and externalization of PS. Additionally, the authors showed that knocking down or pharmacologically blocking TMEM16F suppressed PS exposure and blunted syncytia formation in CoV-2-S positive cells.^134^ In another independent study, the formation of syncytia was also observed in CMs that express CoV-2-S.^123^

Importantly, CoV-2-S not only flags the plasma membrane for fusion but also potently impairs the delicate internal membrane systems that are crucial for maintaining intracellular compartments.^138^ This notion can be inferred from two recent publications. In the work by Braga et al., the authors found that inhibiting the activity of sarco/endoplasmic reticulum Ca^2+^ ATPase (SERCA) abolished CoV-2-S-induced transient Ca^2+^ flux, suggesting that CoV-2-S interferes with SERCA activity to dysregulate the intrinsic Ca^2+^ handling machinery. This observation is especially important for CMs, because CMs have specialized sarcoplasmic reticulum (SR) compartment that serves as Ca^2+^ reservoir, and CM beating is dependent on the synchronized movement of Ca^2+^ into and out of the cell, as well as between the cytosol and SR,^139^ where SERCA is the major ATPase that pumps Ca^2+^ from cytosol into SR.^140^ In line with the study of Braga et al., Han et al. recently reported that CoV-2 infected cardiac pacemaker cells and disrupted the normal Ca^2+^ cycling of these specialized CMs.^117^ Since SR compartment serves as a reservoir to modulate cytosol Ca^2+^ concentration that controls CMs beating frequency and amplitude, future studies are required to define whether CoV-2-S disrupts CM Ca^2+^ handling machinery by impairing the SR structure.

### CoV-2-S as an alarmin to trigger innate immune response

Before binding to ACE2, CoV-2-S needs to be cleaved at the PPC site to expose its receptor binding domain that resides in the S1 subunit. After attaching to host cells, the S2 subunit facilitates virus-host cell membrane fusion and virus entry,^141^ and the cleaved S1 subunit can be shed into body fluid and has been detected in the urine of adult COVID-19 patients.^142^

The direct experimental evidence that CoV-2-S is an inflammatory alarmin comes from three independent studies all of which showed that soluble CoV-2-S1 treatment of THP1 (a human monocytic cell line) cells induced inflammatory response through Toll-like Receptor 4 (TLR4), one of the best-characterized patten recognition receptors (PRRs).^143^, ^144^, ^145^ TLR4 and its downstream signaling cascade consists of a series of adaptor proteins and kinases that lead to the activation of NF-kB complex, a master regulator of inflammation.^146^ Data from my group and other groups have shown that CoV-2-S binds to TLR4 and triggers innate immune responses both *in vitro*^143^^,^^144^ and in *vivo.*^147^ Besides TLR4, Lu et al. have identified 5 ​C-type lectin receptors as myeloid cells (e.g., macrophages) PRRs that bind CoV-2-S and initiate innate immune responses.^148^ These studies thus provide essential molecular evidence that CoV-2-S serves as an alarmin to active innate immune responses (Fig. 3B).

These *in vitro* observations are bolstered by clinical studies that characterize the immune responses triggered by COVID-19 vaccines. mRNA-based COVID-19 vaccines developed by Pfizer BioNTech (BNT162b1) and Moderna (mRNA-1273) have been widely used to reduce CoV-2 infection, and both of these two vaccines use the full-length CoV-2-S coding sequence to induce humoral and cellular immune responses.^149^ Since the time of these COVID-19 mRNA vaccines being authorized for emergency use, a series of clinical and translational studies have been performed to analyze the vaccination-related immune responses. As discussed in the following paragraph, data from these clinical investigations further confirm that CoV-2-S directly activates the innate immune cells.

For traditional vaccines, adjuvants are the essential components that prime the innate immune cells to produce crucial cytokines required for initiating cell (T cells) and humoral (B cells) immune responses.^150^ Although the current COVID-19 mRNA vaccines lack conventional vaccine adjuvants, primary immunization with BNT162B2 is sufficient to activate innate and adaptive immune responses,^151^^,^^152^ which are further increased by the secondary immunization. These clinical studies suggest that mRNA vaccine-encoded CoV-2-S serves as both an adjuvant molecule that activates the innate immune cells and as a pathogen antigen that switches on the adaptive immune responses. Mechanistically, the intro-muscularly injected mRNA vaccines are uptaken and expressed by infiltrated white blood cells (e.g., macrophages),^153^ and the secreted CoV-2-S1 and not the full-length CoV-2-S antigen is released into the bloodstream one-day post-vaccination.^154^ CoV-2-S1 binds to the C-type lectin receptors expressed on the macrophages surface, and this interaction leads to the activation of spleen tyrosine kinase-mediated inflammatory necrosis and the secretion of IL-1β, a pro-inflammatory cytokine that signals native T cell to differentiate into effector memory CD4^+^ T cell.^155^

In summary, CoV-2-S is a putative alarmin that can be recognized by multiple PRRs, and it is able to directly activate innate immune cells, which then synthesize and release cytokines to boost the expansion of adaptive immune cells. Although activation of the innate immune responses is essential for initiating the adaptive immune responses that are pivotal for curtailing CoV-2 infection, over-activation of the innate immune cells may lead to the formation of cytokine storm, which causes collateral damage to the internal organs and underlies the pathogenesis of many COVID-19 related diseases, such as lung and heart injury.^156^

### CoV-2-S activates complement pathway and induces microvascular thrombosis

The complement system is an arm of innate immunity comprised of a large number of plasma proteins that react with one another to opsonize pathogens and eliminate infected host cells. Complement system activation mainly occurs through three proteolytic cascades: a) the classical, b) lectin, and c) alternative complement activation pathways (APC),^157^ all of which converge on the proteolytic processing and activation of C3 convertase.

Being a complicated process involving the polymerization of fibrin and the activation of platelets, blood coagulation is tightly controlled by endothelial cells which express and secret anti-coagulation factors under homeostasis conditions and switch to a pro-thrombotic status when stressed by tissue injury or pathogen invasion.^158^ Previous studies have confirmed the presence of cross-talks between the coagulation and complement systems,^159^ exemplified by the fact that coagulation factors FXa, FXIa cleave C5 and C3 to form active C5a and C3a,^160^ and that C5a and C3a bind to their endothelial receptors to initiate pro-inflammatory signaling events that summon more platelets to the injured sites.^161^

Among the numerous COVID-19 pathological stresses, microvascular thrombosis (a condition where blood clots form in small vessels) is defined as one of the primary causes of lung^162^ and heart^19^ injury. Previous studies and newly published data suggest that CoV-2 infection may activate both the complement and coagulation systems, which will lead to microvascular thrombosis if not managed properly.

Mice deficient in C3 were protected from SARS-CoV infection,^163^ therefore, confirming the involvement of the complement system in the pathogenesis of SARS-CoV-caused acute respiratory distress syndrome. Recently, Yu et al. found that CoV-2-S and not the counterpart of the benign coronavirus OC43 activated APC on the cell surface. Factor H, a component of the complement system, is a negative regulator of the APC and it binds to the heparan-sulfate coated on the cell surface. Because CoV-2-S is known to bind heparan-sulfate,^164^ and adding factor H to the medium mitigated CoV-2-S-induced complement attack in cultured *erythroblast* cells, the authors concluded that CoV-2-S facilitated APC activation on the cell surface by dislodging factor H from heparan-sulfate that anchored on the plasma membrane.^165^

*In vitro* experiments have shown that CoV-2-S is toxic to endothelial cells and that treating endothelial cells with soluble CoV-2-S1 increased their pro-Inflammatory and pro-thrombotic responses,^166^^,^^167^ probably by activating the TLR4/NF-kB pathway.^122^ Furthermore, CoV-2-S1 treatment also caused exuberant C3 and C5b deposition on endothelial cells that further inflames endothelial cells.^167^ Based on these published data, it is reasonable to hypothesize that CoV-2-S1 first coerces the endothelial cells to express pro-inflammatory adhesion molecules and pro-coagulation factors, such as intercellular adhesion molecule (ICAM-1) and von Willebrand factor (vWF), which work together to recruit and activate platelets to form blood clots (Fig. 3C). In addition to directly changing endothelial cell gene expression profiles through the TLR4/NF-kB pathway, CoV-2-S1 may also activate the complement system on the endothelial cell surface, which leads to further endothelium damage and blood clotting.

The current data all fit into a hypothesis that CoV-2 infection damages endothelial cells through CoV-2-S-caused pro-inflammatory responses; however, a crucial question has not been clearly addressed: does CoV-2-S need to engage ACE2 to harm the endothelial cells? One group of studies supports the view that CoV-2-S relies on ACE2 to injure the cultured endothelial cells, because expressing stabilized ACE2 in the endothelial cells enhanced CoV-2-S toxicity^168^; conversely, blocking ACE2 with ACE2-antibody largely saved the endothelial cells from CoV-2-S stress.^167^ In contrast with these two publications, Lei et al. recently examined the endothelial cells derived from human adult and fetal arteries, vascularized tissues and iPSC, and found that only lung endothelial cells express ACE2. Additionally, Lei et al. showed that CoV-2 did not infect iPSC-derived endothelial cells but indeed flamed these cells, probably by activating the TLR4/NF-kB pathway.^122^

As cytokine storm, microvascular injury is another typical pathogenesis of severe COVID-19,^169^ highlighting the emergency of developing therapeutic reagents to prevent or reduce this vascular etiology of COVID-19. To reach this goal, more studies are required to dissect the underlying molecular mechanisms leading to microvascular injury in COVID-19. In the past three years, multiple independent studies indicate that CoV-2-S is the primary CoV-2 viral protein that damages endothelial cells and induces microvascular thrombosis; in the future, more mechanistic studies are required to narrow down the molecular signaling pathways through which CoV-2-S exerts its toxic effects over endothelial cells.

### CoV-2-S as a putative superantigen (SAg)

Over the past three years, numerous clinical studies have confirmed that dysregulated innate and adaptive immune responses are central to the pathogenesis of COVID-19.^170^ As aforementioned, CoV-2-S may serve as an alarmin to activate innate immune responses. Here by introducing the superantigen (SAg) concept, I will discuss the possibility of CoV-2-S being a SAg to over-activate the adaptive immune system.

T lymphocytes are one of the two arms of adaptive immune cells, and the maturation and activation of T cells require robust antigen selection processes.^171^ During a conventional antigen-recognizing process, the antigen needs to be captured and processed by antigen-presenting cells (e.g., dendritic cells), and the processed antigen fragments are bound by Major Histocompatibility Complex class II (MHC class II) and presented to naive T cells through T cell receptors (TCRs); the formation of MHC II-antigen-TCR complex then leads to T cells proliferation and activation.

Different from conventional antigens, a SAg does not need to be processed by antigen-presenting cells, and the intact SAg directly interacts with both class II MHC molecules and the Variable region of the T cell receptor β chain (Vβ), thus skipping the antigen selection process and quickly activates a large amount of T cells (up to 20%).^172^ One of the best-known SAgs is bacteria-derived staphylococcal enterotoxin B (SEB), a toxic protein that causes intense T-cell proliferation and cytotoxicity.^173^

COVID-19 rarely causes pneumonia in children; however, multisystem inflammatory syndrome in children (MIS-C) has been observed as another category of COVID-19-related diseases, manifesting as persistent fever and hyperinflammation in multiple organs, such as the heart, gastrointestinal tract, and kidney.^174^ A superantigen hypothesis has been proposed to explain the pathophysiology of MIS-C^175^: CoV-2-S directly interacts with TCR β chain variable domain through its putative superantigen motif (T_678_ to Q_690_), therefore activating T cells and triggering hyperactive adaptive immune responses^175^ (Fig. 3D).

The TCR β chain gene locus contains a cluster of 52 ​V_β_ gene segments, which rearrange during T-cell development to form complete V-domain exons.^171^ Each SAg is specific to one or a few of the V_β_ gene segments, and so a SAg can stimulate 2%–20% of the total T cells. If CoV-2-S is a SAg, the V_β_ gene segments of the T cells from COVID-19 patients should be different from healthy control donors. Indeed, by analyzing the next-generation immunosequencing data derived from mild/moderate and severe COVID-19 patients, Cheng et al., found that the principal components of the TCR β-chain variable gene (TRBV) repertoires corresponding to mild/moderate COVID-19 patients clustered apart from those with severe COVID-19 symptoms. Until now, six TCR V_β_ gene segments have been shown to be enriched in severe COVID-19 patients, including V_β_ 5–6, V_β_ 13, V_β_ 14, V_β_24-1^175^ and V_β_11-2,^176^,^177^ and in silico analysis predicted that TCRs containing these V_β_ segments interact with CoV-2-S.^175^^,^^177^

To test whether CoV-2-S has SAg activity in experimental systems, Amormino et al. assessed the SAg activity of the CoV-2-S in cultured Jurkat T cell line and primary CD4^+^ T cells, and their results showed that CoV-2-S did exhibit an intrinsic SAg-like activity in these cultured T cells.^178^ Although these *in vitro* data are at odd with the CoV-2-S/superantigen hypothesis, it is still possible that CoV-2-S synergies with other endotoxins to act as a superantigen complex. For example, CoV-2-S has been shown to interact with lipopolysaccharide (LPS),^179^ a bacteria-derived endotoxin. Delivering CoV-2-S itself into mice did not cause significant inflammation; however, CoV-2-S robustly increased the inflammatory effect of a low dose LPS,^179^^,^^180^ thus supporting the notion that CoV-2-S may combine with other molecules to behave as a SAg complex.^181^ In consideration that severely ill COVID-19 patients have high levels of serological markers for disrupted intestinal barrier integrity and microbial translocation,^182^ it is valuable to further test this CoV-2-S/LPS superantigen complex hypothesis in the near future.

Although being attractive, the CoV-2-S/superantigen hypothesis needs to be further developed, and the generation of suitable animal models is essential for reaching this goal. To understand more about whether and how the CoV-2 virus triggers superantigen effects, the audiences are encouraged to read a recently published review.^181^

## Future directions

CoV-2 is a new zoonotic virus threatening our daily life. In the past three years, human society has been struggling to fight this virus and has developed new tools for treating COVID-19. Nevertheless, CoV-2 is rapidly evolving and new immune-evading variants are quickly emerging. Due to its essential role in infection and virus packaging, CoV-2-S is the mostly mutated antigen during CoV-2 evolution, therefore the mutations in CoV-2-S are used to define variants of concerns.^183^ Clearly, characterizing CoV-2-S biochemical properties is critical to define the pathogenic differences between different variants, which will provide solid foundations for developing new therapeutics against COVID-19.

Recently published data have confirmed that Omicron CoV-2 has reduced pathogenicity compared to its ancestors^184^, ^185^, ^186^ and pinpointed the inefficient S1/S2 cleavage as the major reason accounting for Omicron CoV-2 virulence attenuation,^184^^,^^185^ further highlighting the importance of studying the pathological roles of CoV-2-S in COVID-19.

CoV-2-S mRNA vaccine has been widely used to combat COVID-19. Although generally safe, one of the major adverse effects of the CoV-2-S mRNA vaccine is causing myocarditis in rare cases. A recent study compared the immune responses between individuals who developed post-vaccine myocarditis and those of healthy vaccinated control subjects, and the results showed a modest increase in cytokine production and markedly elevated levels of free (unbound by antibodies) full-length spike protein in the plasma of post-vaccine myocarditis patients, whereas this full-length spike protein was not detectible in the healthy vaccinated individuals.^187^ These new results raised a concern about whether CoV-2-S is the culprit that directly causes myocarditis, thus requiring further investigations to elucidate the toxicity effects of CoV-2-S in the cardiovascular context.

In summary, fully understanding the pathogenesis of COVID-19 is an urgent medical need. Since CoV-2-S plays essential roles in the pathogenesis of COVID-19, it is essential and rewarding to set up the novel *in vitro* and *in vivo* experimental platforms to define how CoV-2-S disrupts host cell structure and function, which will inform the efforts of developing safe and efficient therapeutics for blocking CoV-2 infection and reducing COVID-19 symptoms.

## Submission statement

This work has not been published previously and it is not under consideration for publication elsewhere. This manuscript is approved by the author and by the responsible authorities where the work was carried out. If accepted, this work will not be published elsewhere including electronically in the same form, in English or in any other language, without the written consent of the copyright-holder.

## Funding

Z.L. was supported by 10.13039/100000050NHLBI (Grant number: R01HL146810).

## Conflict of interest

The author declares that potential competing interests do not exist.
